# SpyTag/SpyCatcher display of influenza M2e peptide on norovirus-like particle provides stronger immunization than direct genetic fusion

**DOI:** 10.3389/fcimb.2023.1216364

**Published:** 2023-06-22

**Authors:** Vili Lampinen, Stina Gröhn, Saana Soppela, Vesna Blazevic, Vesa P. Hytönen, Minna M. Hankaniemi

**Affiliations:** ^1^ Protein Dynamics, Faculty of Medicine and Health Technology, Tampere University, Tampere, Finland; ^2^ Virology and Vaccine Immunology, Faculty of Medicine and Health Technology, Tampere University, Tampere, Finland; ^3^ Vaccine Development and Immunology/Vaccine Research Center, Faculty of Medicine and Health Technology, Tampere University, Tampere, Finland; ^4^ Fimlab Laboratories, Tampere, Finland

**Keywords:** influenza, norovirus, SpyCatcher, virus-like particle, norovirus (NoV), conjugation, fusion protein, cell mediated and humoral immunity

## Abstract

**Introduction:**

Virus-like particles (VLPs) are similar in size and shape to their respective viruses, but free of viral genetic material. This makes VLP-based vaccines incapable of causing infection, but still effective in mounting immune responses. Noro-VLPs consist of 180 copies of the VP1 capsid protein. The particle tolerates C-terminal fusion partners, and VP1 fused with a C-terminal SpyTag self-assembles into a VLP with SpyTag protruding from its surface, enabling conjugation of antigens via SpyCatcher.

**Methods:**

To compare SpyCatcher-mediated coupling and direct peptide fusion in experimental vaccination, we genetically fused the ectodomain of influenza matrix-2 protein (M2e) directly on the C-terminus of norovirus VP1 capsid protein. VLPs decorated with SpyCatcher-M2e and VLPs with direct M2 efusion were used to immunize mice.

**Results and discussion:**

We found that direct genetic fusion of M2e on noro-VLP raised few M2e antibodies in the mouse model, presumably because the short linker positions the peptide between the protruding domains of noro-VLP, limiting its accessibility. On the other hand, adding aluminum hydroxide adjuvant to the previously described SpyCatcher-M2e-decorated noro-VLP vaccine gave a strong response against M2e. Surprisingly, simple SpyCatcher-fused M2e without VLP display also functioned as a potent immunogen, which suggests that the commonly used protein linker SpyCatcher-SpyTag may serve a second role as an activator of the immune system in vaccine preparations. Based on the measured anti-M2e antibodies and cellular responses, both SpyCatcher-M2e as well as M2e presented on the noro-VLP via SpyTag/Catcher show potential for the development of universal influenza vaccines.

## 1 Introduction

Influenza has been, and still is, one of the most prevalent microbial diseases tormenting humankind. Though an influenza infection rarely hospitalizes healthy adults, it can lead to serious and even fatal complications, especially in the young and elderly. On a global scale, WHO estimates between 3 and 5 million infections with serious complications and 290 000–650 000 deaths due to influenza every year (https://www.who.int/news-room/fact-sheets/detail/influenza-(seasonal); accessed 28.4.2023). Influenza is widespread among mammals and birds, and due to its segmented genome, influenza occasionally goes through a genetic shift between strains from different host species that allows the hybrid strain unparalleled transmissibility, causing pandemics (Wolfe et al., 2007).

Effective vaccines against influenza exist, but due to the fast evolution rates of the RNA virus, these need annual renewal to keep up. A long-lasting, universal influenza vaccine has been the target of heavy research efforts for decades, but none have reached the clinic yet. The most promising universal influenza vaccine candidates direct the immune response against conserved parts of the influenza virus. The ectodomain of Matrix 2 proton channel is only 24 amino acid residues long, but it is >90% conserved across different influenza strains (Ebrahimi and Tebianian, 2011), making it an attractive target for a universal influenza vaccine.

Short peptides, like M2e, are not very immunogenic by themselves, so they must be attached to an immunogenic carrier, such as a virus capsid protein. The spontaneous assembly of viral proteins into virus-like particles (VLPs) enables multivalent presentation of target antigens on the VLP surface, which can increase the efficiency of the B-cell response against small peptides, independent of T-cells (Chackerian et al., 2008). Most VLPs have diameters (10–200 nm) that are optimal for uptake by antigen-presenting cells and for direct drainage into the lymphatic system (Bachmann and Jennings, 2010).

Our previous studies on norovirus-like particles (noro-VLPs) have revealed that they are particularly robust and easy to modify, produce and store (Koho et al., 2015; Lampinen et al., 2021). Noro-VLPs consist of 180 repeats of the single capsid protein, VP1 (Prasad et al., 1994), that assemble to form the noro-VLP so that C-terminal extensions are presented on the particle surface (Koho et al., 2015). In earlier experiments, we utilized this by genetically fusing SpyTag on the noro-VP1 C-terminus and then covalently conjugating the SpyTags with SpyCatcher-fused influenza M2e peptides (Lampinen et al., 2021; Heinimäki et al., 2022). SpyTag and SpyCatcher are two halves of a split protein system that spontaneously reforms *via* a covalent isopeptide bond upon contact in a variety of conditions (Zakeri et al., 2012). The system has been used successfully in many labs for decoration of VLPs for vaccination (e.g. (Brune et al., 2016; Thrane et al., 2016; Rahikainen et al., 2021)).

**Figure 1:**
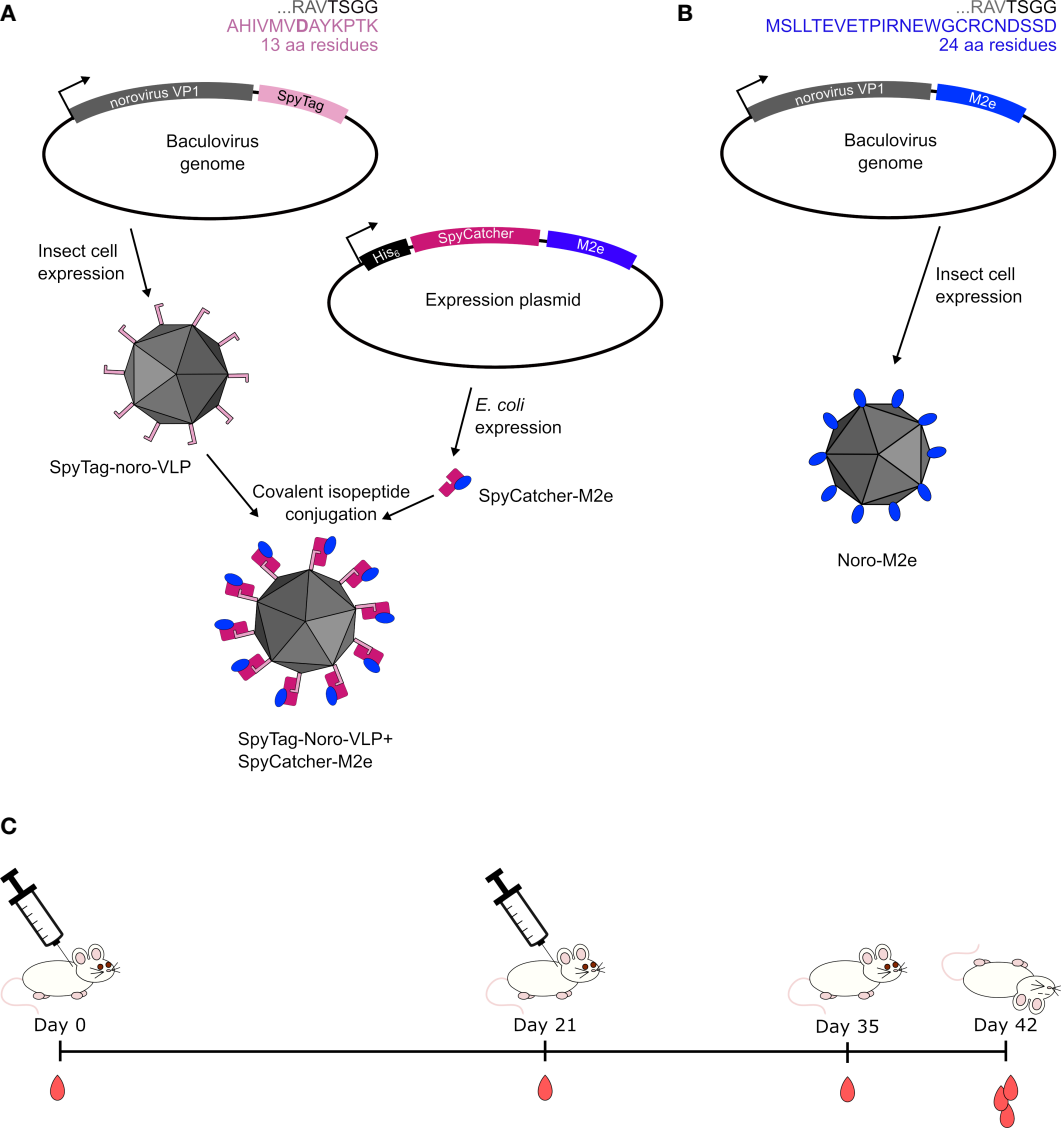
Design of vaccines used in this study. **(A)** Earlier studies (Lampinen et al., 2021) show that when a 13-amino-acid SpyTag is genetically fused to the C-terminus of noro-VP1, the tag is available on the outside of the assembled norovirus-like particle. SpyCatcher with a fused protein antigen can be separately expressed in a bacterial system and mixed with SpyTag-noro-VLP to yield decorated VLP. **(B)** A 24-amino-acid M2e peptide was genetically fused to the C-terminus of noro-VLP for producing noro-VLPs displaying the M2e peptide in insect cells. **(C)** We injected these vaccine preparations twice in BALB/c mice with their control groups and compared the produced immune responses at different time points in the study.

Previously, we immunized mice with unadjuvanted SpyCatcher-M2e-decorated noro-VLP, but few anti-M2e antibodies were formed (Heinimäki et al., 2022). This led us to suspect that the *Streptococcus pyogenes* bacteria-derived SpyCatcher may mask the small M2e peptide from the immune system, so here, we produced a form of noro-VLP that presents influenza M2e as a direct genetic fusion on its C-termini (**Figure 1**). We immunized mice subcutaneously with the genetically fused M2e-noro-VLP, SpyCatcher-M2e alone and, also, SpyCatcher-M2e-decorated noro-VLP in doses comparable to the earlier experiments with and without an Al(OH)_3_ adjuvant. The present study shows a dramatic enhancement to the anti-M2e immune response of the SpyCatcher-M2e-decorated noro-VLP vaccine candidate tested before, apparently due to the addition of alum adjuvant. The group immunized with adjuvanted soluble SpyCatcher-M2e also generated high titers of anti-M2e antibodies, making both vaccines attractive candidates for further development of universal influenza vaccines. We were able to produce and purify the genetically fused M2e-noro-VLP, but mice immunized with it produced less anti-M2e antibodies compared to SpyCatcher-M2e immunized animals. On the other hand, noro-VLP-displayed M2e showed higher cellular responses compared to simple SpyCatcher-M2e fusion, which supports further studies on noro-VLP presented influenza M2e peptide.

## 2 Results

### 2.1 Noro-VLP tolerates direct fusion of M2e

**Figure 2:**
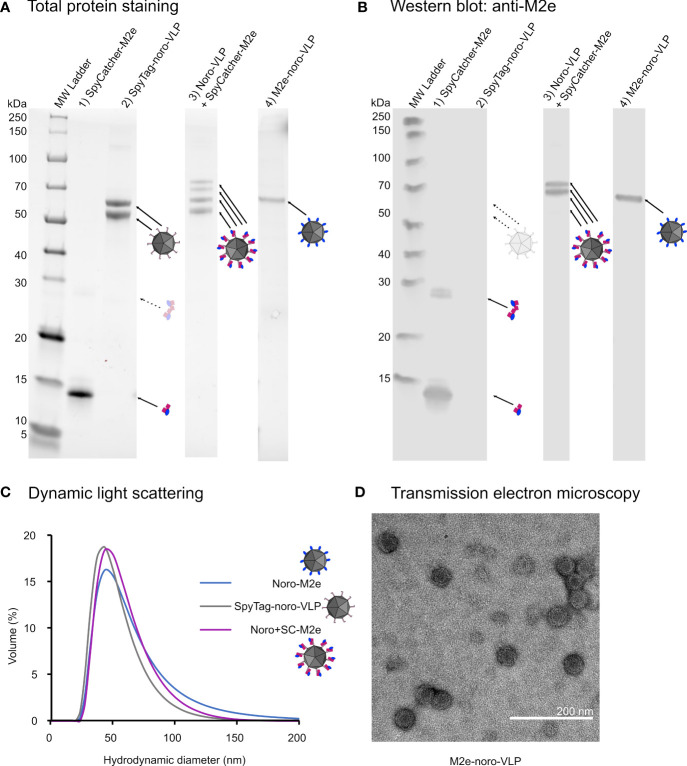
Characterization of the produced vaccine antigens. **(A)** An image of a Stainfree total protein gel with approximately 1 µg of total protein loaded in each well. SpyTag-noro-VP1 appears as a double band due to an N-terminal truncation, which is not seen in M2e-noro-VLP. Upon conjugation, some of the SpyTag-noro-VLP double band moves upwards by the size of the conjugated SpyCatcher-M2e. **(B)** The same gel blotted onto membrane for detection by M2 antibody. Only the conjugated noro-VLP double band is identified by the M2 antibody. A small amount of SpyCatcher-M2e is in dimeric form on the gel, despite boiling the sample. **(C)** Dynamic light scattering results of the hydrodynamic sizes of different noro-VLPs from the volume-based distribution. The mean peak sizes are 49 nm for SpyTag-noro-VLP, 54 nm for noro-VLP+SpyCatcher-M2e and 58 nm for M2e-noro-VLP. **(D)** A representative image of M2e-noro-VLP in transmission electron microscopy.

We produced SpyTag-noro-VLP in insect cells as described previously (Lampinen et al., 2021), but utilized size-exclusion chromatography as a new method for removing the residual baculovirus (**Supplementary Figure 1**). Compared to the anion exchange method used earlier for this purpose, we were able to almost double the VLP production yield from 10–30 to 40–80 mg/L. After setting up the production and purification methods for the SpyTag-noro-VLP, the same protocol was utilized to produce and purify a noro-VLP with genetically fused influenza M2e peptide in its C-terminus. In gel electrophoresis, M2e-noro-VLP (calculated mass 62 150 Da) was detected as a clear, single band slightly larger than SpyTag-noro-VLP (60 867 Da) in size, as expected (**Figure 2A**). M2e-noro-VLP was produced with yields of approximately 25 mg/L with this protocol, which is comparable to the SpyTag-noro-VLP yields reported with anion exchange purification (Lampinen et al., 2021).

We conjugated SpyCatcher-M2e on SpyTag-noro-VLP as described earlier, now with a conjugation efficiency of 24%. The monoclonal anti-influenza-M2 antibody recognized the SpyCatcher-fusion of M2e covalently bound to noro-VLP as well as the genetic fusion between M2e and norovirus-VP1 (**Figure 2B**). Dynamic light scattering confirmed that M2e-fused noro-VP1 can assemble into homogenic particles (hydrodynamic diameter=54 nm; PdI=0.178 ± 0.008) that seem slightly larger than SpyTag-noro-VLPs (49 nm; PdI=0.118 ± 0.016) (**Figure 2C**). According to negative staining transmission electron microscopy, M2e-noro-VLP is indistinguishable in morphology from wild type noro-VLPs (**Figure 2D**) (Jiang et al., 1992; Ausar et al., 2006; Lampinen et al., 2021), which is expected given that the small fusion partner is positioned into a valley between protruding domains, based on the noro-VLP crystal structure (RCSB PDB ID: 1IHM (Prasad et al., 1999)). All recombinant proteins used in vaccinations were purified to >95% purity and confirmed to contain<700 pg dsDNA and<1.5 EU endotoxins per µg of vaccine antigen, meeting the criteria set for preclinical experimental vaccines.

The influenza M2e peptide (24 aa) is almost double in size compared to SpyTag (13 aa), so we wanted to see if attaching such a large fusion partner to noro-VLP would affect its thermal stability. Differential scanning fluorimetry (DSF, a.k.a. thermofluor) analysis of SpyTag-noro-VLP and M2e-noro-VLP side by side showed that M2e-noro-VLP disassembly and unfolding indeed begins at a lower temperature compared to SpyTag-noro-VLP (**Supplementary Figures 2A, B**). Its melting temperature (Tm) of 53.2 ± 0.5°C is 14.3°C lower than that of SpyTag-noro-VLP (67.5 ± 0.6°C) (n=3). We observed no changes in the unfolding profile and melting point of either SpyTag-noro-VLP or M2e-noro-VLP in salt concentrations of 60, 150 and 300 mM. However, despite significant destabilization as compared to SpyTag-noro-VLP, the thermal stability of M2e-noro-VLP is sufficient to tolerate long storage periods in typical conditions and no signs of unfolding were observed upon storage for several months at +4°C (data not shown). The melting curve of M2e-noro-VLP obtained from DSF is broadened and perhaps hints at a two-step unfolding mechanism. This model was further supported by differential scanning calorimetry (DSC) analysis, where M2e-noro-VLP showed a clear biphasic unfolding trace. The lower Tm_1_ (67.18 ± 0.07°C) of M2e-noro-VLP was 4.5 degrees lower than the Tm_1_ measured for wild type noro-VLP (71.68 ± 0.16°C) (**Supplementary Figures 2C, D**). Accordingly, DSC showed a 1.0°C reduction between the Tm_2_ of M2e-noro-VLP and the Tm_2_ of wild type noro-VLP (74.07 ± 0.05 vs. 75.07 ± 0.11°C).

### 2.2 M2e immune responses are strengthened by protein fusion and adjuvant

**Figure 3:**
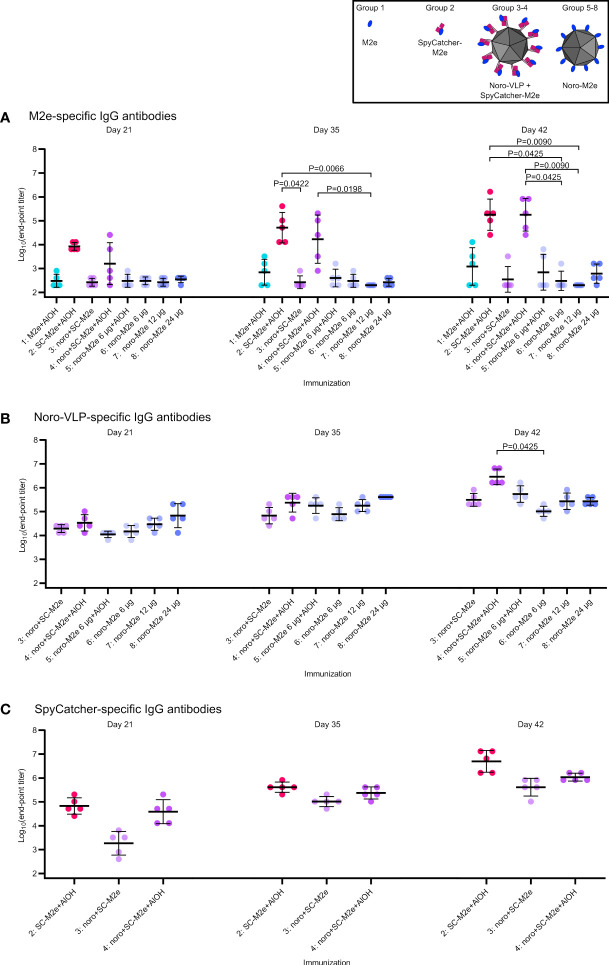
Total IgG responses at different time points. Log10 transformations of IgG antibody end-point titers against M2e peptide **(A)**, noro-VLP **(B)** or SpyCatcher **(C)**, as assessed in ELISA wells coated with the peptide or protein indicated above each graph. Mean titers are represented by the thick line ± standard deviation. P values are shown for groups with a difference with P<0.05, determined by Dunn’s test. Each dot represents a single mouse. Undetectable antibody levels were denoted with the titer 200 (half of the lowest dilution assessed).

To assess the influence of different methods of influenza M2e presentation on its immunogenicity, mice were immunized with various antigen compositions. We then assessed the levels of IgG antibodies formed against the peptide, the noro-VLP carrier and SpyCatcher linker protein at different time points in the animal experiment. At day 0, we detected no antibodies against any of the tested antigens (data not shown). Overall, IgG levels increased with time, especially after the second immunization at day 21 (**Figure 3**).

To evaluate if the vaccination could be potentiated with adjuvant, vaccination group 4 (G4: M2e presented on noro-VLP *via* SpyCatcher), was given ¼ of the M2e dose compared to our previous experiment (Lampinen et al., 2021; Heinimäki et al., 2022), but the vaccine was adsorbed on Al(OH)_3_ adjuvant. This resulted in a significant increase in the amount of anti-M2e antibodies after a single immunization compared to non-adjuvanted vaccine. On day 42, the geometric mean end-point titer (GMT) of G4 was 1.8*10^5^, compared to a titer of 100 obtained without adjuvant (Heinimäki et al., 2022). Surprisingly, adjuvanted SpyCatcher-M2e was equally good at raising anti-M2e antibodies as M2e presented on noro-VLP *via* SpyCatcher (also showing GMT of 1.8*10^5^). In contrast to our initial hypothesis, M2e-noro-VLP was not nearly as effective in directing the immune response towards the presented M2e peptide as SpyCatcher-conjugated M2e on noro-VLP or even SpyCatcher-M2e alone. The adjuvanted M2e-noro-VLP group 5 reached a GMT of 700, while the mice vaccinated with unadjuvanted M2e-noro-VLP had almost undetectable levels of anti-M2e antibodies (G6: 300, G7: 200, G8: 610).

Very high titers (>1*10^5^) of anti-noro antibodies were formed by all noro-VLP-containing vaccines after two immunizations, regardless of the presence of M2e antigen, SpyCatcher or adjuvant. Therefore, it seems likely that display of foreign antigens does not compromise the potential of the noro-VLPs as a vaccine against norovirus, as shown earlier in a similar context (Heinimäki et al., 2022). Conversely, SpyCatcher response was 5-fold stronger when presented without noro-VLP (G2: 5.0*10^6^ vs. G4: 1.1*10^6^). Furthermore, anti-noro response seemed less dependent on adjuvant as compared to M2e response, but still, increases in the antibody response were observed in groups with Al(OH)_3_ compared to groups without it (G4: 2.9*10^6^ vs. G3: 3.1*10^5^ and G5: 5.4*10^5^ vs. G6: 1.0*10^5^).

We previously used a noro-VLP dose of 43 µg to immunize mice with antigen-decorated noro-VLP (**Table 1**). To find the optimal dosing for strong immunity, we evaluated the antibody response with different antigen doses. Immunization with an 11-µg dose of noro-VLP (G7: 2.7*10^5^) showed a trend of increasing anti-noro antibodies over those obtained with a dose of 6 µg (G6: 1.0*10^5^), but no large difference could be seen when increasing noro-VLP dosage over 11 µg (G3: 3.2*10^5^ and G8: 2.7*10^5^).

**Table 1:** Vaccination groups and doses used in this study and in two related studies.

Group	Vaccine	Total protein dose (µg)	M2e dose (µg)	Noro-VLP dose (µg)	SpyCatcher dose (µg)
1	M2e+Al(OH)_3_	1.06	1.1	0.0	0.0
2	SC-M2e+Al(OH)_3_	1.5	0.3	0.0	1.2
3	noro+SC-M2e	31.0	0.3	28.6	1.2
4	noro+ SC-M2e+Al(OH)_3_	31.0	0.3	28.6	1.2
5	noro-M2e+Al(OH)_3_	6.0	0.3	5.7	0.0
6	noro-M2e	6.0	0.3	5.7	0.0
7	noro-M2e	12.0	0.5	11.4	0.0
8	noro-M2e	24.0	1.1	22.8	0.0
9	Buffer+Al(OH)_3_	0	0	0	0
Lampinen et al., 2021	Noro+SC-M2e	50	1.1	42.8	4.8
Heinimäki et al., 2022	M2e+Al(OH)_3_	50	50	0	0

**Figure 4:**
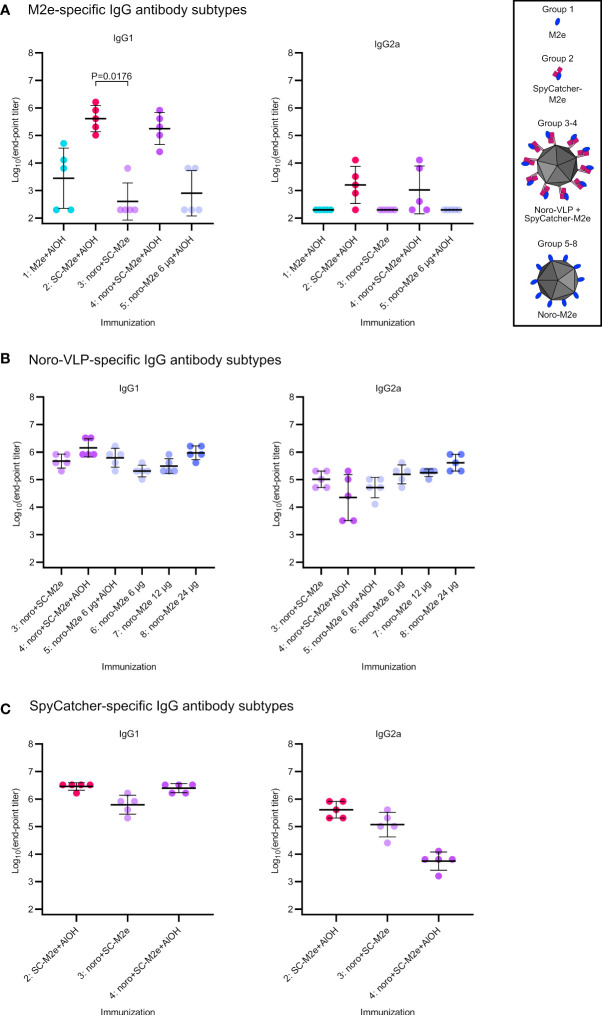
IgG antibody subtype comparison on day 42 (sacrifice). Log10 transformations of IgG antibody end-point titers against M2e peptide **(A)**, noro-VLP **(B)** or SpyCatcher **(C)**, as assessed in ELISA wells coated with the peptide or protein indicated above each graph. Here, only mice with detectable total IgG antibodies were measured, unmeasured mice were denoted with the titer 200 and unmeasured groups were omitted from the graphs. Mean titers are represented by the thick line ± standard deviation. P values between groups are shown for pairs with a difference in means with P<0.05, determined by Dunn’s test. Each dot represents a single mouse. Undetectable antibody levels were denoted with the titer 200 (half of the lowest dilution assessed).

**Figure 5:**
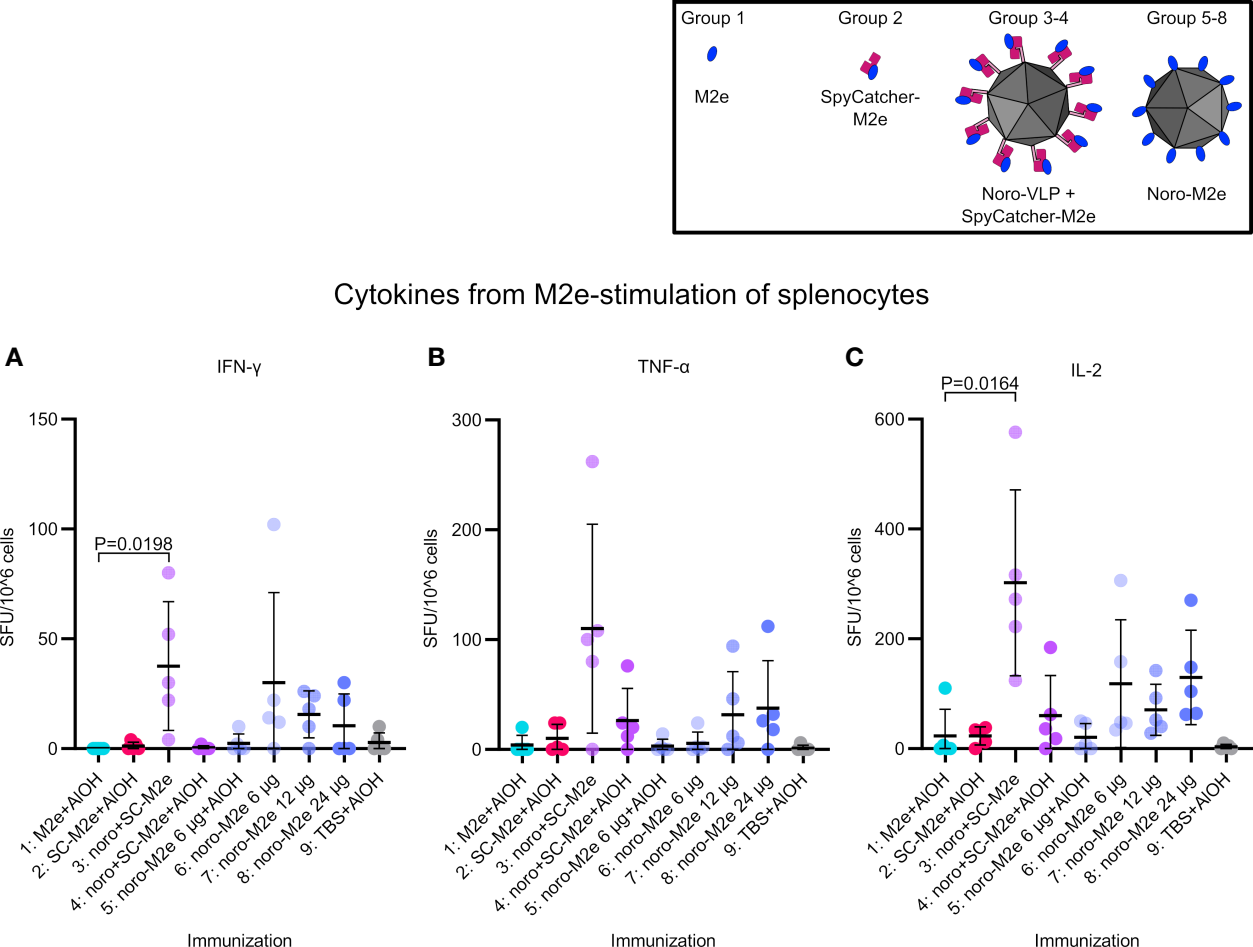
Interferon gamma **(A)**, tumor necrosis factor alpha **(B)** and interleukin-2 **(C)** levels obtained by stimulating splenocytes extracted from vaccinated mice. Mean positive cell counts are presented as thick lines ± standard deviation. P values between groups are shown for pairs with a difference in means with P<0.05, determined by Dunn’s test. Each dot represents a single mouse.

The doses of M2e, noro-VLP and SpyCatcher are calculated by comparing its mass to other proteins in the preparation and considering conjugation efficiency where applicable. A detailed explanation of dose calculation is provided in Materials and Methods 4.3. Highlight colors refer to the study group colors used in **Figures 3**–**5**.

While SpyCatcher/Tag system has been utilized widely, to our knowledge, no one has evaluated the immunogenicity of the SpyTag peptide. Even in the presence of adjuvant, GMT of IgG response against SpyTag was <1*10^3^, and no cellular response was observed, either (**Supplementary Figure 3**).

Detectable IgG responses against M2e, SpyCatcher and noro-VLP antigens were also subtyped. As a generalization, IgG1 antibodies are associated with Th2-type antibody-mediated response, while IgG2a antibodies are more related to Th1-type cellular immune response (Mosmann and Coffman, 1989). Formulating antigens with Al(OH)_3_ boosted IgG1 responses at the expense of IgG2a responses for all the studied antigens (**Figure 4**), as expected (Brewer et al., 1996). Adjuvant-free antibody responses were slightly biased towards IgG1.

### 2.3 Presence of noro-VLP strengthens the cellular immune response

We analyzed the cytokine responses (IFN-γ, TNF-α, IL-2) elicited by the vaccine candidates by FluoroSpot assay (**Figure 5**). The numbers of M2e-reactive IFN-γ- and IL-2-secreting splenocytes in mice immunized with SpyCatcher-M2e-decorated noro-VLPs (G3: arithmetic means of 38 and 302, respectively) were significantly higher than those measured for the adjuvanted M2e peptide group (G1: 0 and 23). However, formulating SpyCatcher-M2e-decorated noro-VLPs with Al(OH)_3_ adjuvant decreased the cytokine responses (IFN-γ (G3 vs. G4): 38 vs. 0, TNF-α (G3 vs. G4): 110 vs. 26, IL-2 (G3 vs. G4): 302 vs. 60). The presence of noro-VLP led to a trend of improved cytokine responses in comparison to group 1 immunized with M2e peptide and Al(OH)_3_ formulation, whose means cell numbers were 0, 4 and 23 for IFN-γ, TNF-α and IL-2, respectively. Negligible cytokine secretion could be detected in the group of mice immunized with Al(OH)_3_-formulated SpyCatcher-M2e when stimulated with M2e (**Figure 5A**). Both IFN-γ and IL-2 responses were negligible in FluoroSpot analyses when using SpyCatcher or noro-VLP as stimulants (**Supplementary Figure 4**). We did observe some TNF-α signals here, but these appeared also in the buffer control group splenocytes, possibly related either to reactions of innate immune cells in the spleen to the stimulants or to unspecific inflammation responses due to handling of the splenocytes.

## 3 Discussion

Attempts to use M2e peptide to obtain protective immunization against various influenza strains stems from the high conservation rate of the peptide. However, it has turned out somewhat challenging to induce strong immunization with this antigen. In our previous study, we used influenza M2e peptide displayed on noro-VLP *via* SpyCatcher/SpyTag conjugation to immunize mice *via* the intramuscular route, but these mice generated only low levels of anti-M2e antibodies. To assess if the SpyCatcher linker, originating from *S. pyogenes*, was dominating the immune response over the small M2e peptide, we now eliminated the linker from the vaccine candidate by preparing M2e presented on noro-VLP *via* genetic fusion. The resulting immune response was compared to that obtained with M2e SpyCatcher-conjugated on Spy-tagged noro-VLP (**Figure 1**).

We produced SpyTag-noro-VLP and M2e-noro-VLP in insect cells with the help of baculovirus vector as reported earlier (Lampinen et al., 2021), but now utilized size exclusion chromatography (SEC) instead of ion exchange for the separation of residual baculovirus. The method worked well, increasing noro-VLP production yield up to 2-fold and separating baculovirus (200–400 nm in length (Boucias and Pendland, 2012)) from the smaller norovirus-like particle (~40 nm (White et al., 1997; Lampinen et al., 2021)) efficiently. In Western blot analysis of SEC elution fractions, baculovirus transmembrane protein gp64 was observed both before and after the noro-VLP peaks, probably reflecting intact baculovirus in the early fractions and soluble gp64 released from disassembled baculovirus eluting after noro-VLP. The main perk of SEC over ion exchange chromatography is that modifications made on the VLP surface do not affect the purification parameters. For example, replacing SpyTag with M2e on noro-VP1 C-terminus may have changed the charge of the VLP surface enough that ion exchange elution buffer would have required optimization. However, for large-scale manufacturing of noro-VLPs, optimization of ion exchange chromatography protocol would be worth the trouble for being more scalable than SEC.

Even though M2e is almost double in size as a fusion partner compared to SpyTag, noro-VLP self-assembled efficiently despite the direct C-terminal fusion of M2e. We were not quite able to reach the superb yields of SpyTag-noro-VLP (40–80 mg/L), but with an optimized purification protocol involving SEC, we obtained >95% pure M2e-noro-VLP with yields exceeding 20 mg/L. In electrophoresis, we noted that M2e-noro-VP1 appears as a clear, single-band protein, even though our (Koho et al., 2015; Lampinen et al., 2021) and many other labs’ (Jiang et al., 1992; White et al., 1997; Bertolotti-Ciarlet et al., 2002; Tan et al., 2004) noro-VLP preparations were double-banded. This reflects the use of different baculovirus genomes in VLP expression (Lampinen et al., unpublished results). The thermal stability of M2e-noro-VLP (Tm=53°C according to DSF) was lower as compared to SpyTag-noro-VLP (Tm=67°C) and wild type noro-VLP (Tm=68°C) (Lampinen et al., 2021), but still sufficient for good storage stability in typical conditions. We encountered no deterioration of M2e-noro-VLP upon storage for months at +4°C, which is a clear advantage for a vaccine candidate. The melting curve of M2e-noro-VLP measured with DSC suggested a 2-step unfolding mechanism. This was also observed for wild type noro-VLP (WT). In line with DSF, the measured thermal transitions were lower for the particle with M2e fusion partner (Tm_1_(M2e)=67°C, Tm_1_(WT)=72°C; Tm_2_(M2e)=74°C, Tm_2_(WT)=75°C). Based on these stability studies and earlier studies on the disassembly of wild type noro-VLPs (Ausar et al., 2006), we suggest an unfolding model where the M2e peptide destabilizes the noro-VLP particle in a way that makes it disassemble at a lower temperature compared to VLP without a peptide fusion, producing the distinct Tm_1_ peak. The melting temperature of the Tm_2_ peak is very close to that of wild type noro-VLP and could be related to unfolding of disassembled VP1 monomers, where a terminal fusion peptide is likely to have only a minor contribution. Confirming the model would require further biophysical studies.

SpyCatcher-M2e was conjugated on the outside of SpyTag-noro-VLPs and conjugation efficiency was measured by mobility shift assay using polyacrylamide gel electrophoresis (PAGE), as described earlier (Lampinen et al., 2021). Here, we observed a conjugation efficiency of 24%, even though SpyCatcher-M2e was added in excess to the reaction. In our previous study, we reported an efficiency of 50% for the conjugation. This phenomenon was evaluated with multiple control experiments, where we noticed that storing mixtures of sodium dodecyl sulphate (SDS) -denatured SpyCatcher and noro-VLP for prolonged periods before PAGE analysis led to overestimation of conjugation efficiency (data not shown). Therefore, it appears that SpyCatcher can renature to some extent in the presence of SDS, and thus the conjugation may proceed even after SDS/heat treatment. In favor of this hypothesis, previous studies have shown that certain heat-denatured proteins are able to refold slowly in the presence of SDS (Kaspersen et al., 2017). Future studies should assess the exact conditions required for SpyCatcher refolding and its dynamics.

Even though M2e-noro-VLP was easy to produce and proved structurally stable, it was not very effective in inducing immune responses against M2e in mice. The strong M2e-response elicited by SpyCatcher-M2e (group 2) and the poor immunological performance of M2e-noro-VLPs proves our initial hypothesis wrong, as SpyCatcher does not seem to mask M2e from the immune system at all. Two mice in the adjuvanted M2e-noro-VLP group (group 5) reached titers of >3*10^3^, but then again, three mice had undetectable anti-M2e antibody levels at day 42, indicating that this vaccine preparation is too poorly immunogenic to generate a repeatable immune reaction. M2e-noro-VLP fared slightly better in generating cell-mediated immune responses against M2e than the SpyCatcher-M2e group, but conversely, for cell responses the presence of Al(OH)_3_ was detrimental. Higher antibody titers were reported with similar genetic fusions of M2e peptide on hepatitis B VLPs (Neirynck et al., 1999), bacteriophage AP205 VLPs (Tissot et al., 2010) and on norovirus P-particles (Xia et al., 2011). The C-termini of noro-VLPs are located in valleys between the surrounding protruding domains, which positions the M2e peptides (equipped with only a short linker in our construct) between noro-VLP protrusions, potentially limiting the access of large proteins. In future studies, this could be potentially circumvented by fusing multiple peptides in succession to a VLP carrier, as described in (Kim et al., 2013; Lee et al., 2018; Lee et al., 2019) or by separating M2e from noro-VP1 C-terminus by an enlengthened linker like the one introduced recently (Boonyakida et al., 2023). Boonyakida et al. used the linker to allow more space between noro-VP1 C-terminus and SpyTag, enhancing conjugation rates for SpyCatcher-fused proteins. Accordingly, a longer linker between noro-VP1 and M2e and/or multiple repeats of M2e may help to increase the anti-M2e immune responses in future experiments, but this can be expected to further destabilize noro-VLP and possibly disturb VLP assembly, and therefore, it can lead to lower yields.

Using SpyTag/Catcher-mediated M2e peptide display on noro-VLPs probably leaves SpyCatcher in between protruding domains but most likely carries M2e itself to the outside of noro-VLP. By formulating the vaccines with Al(OH)_3_ adjuvant, we were able to generate biologically relevant anti-M2e antibody levels in groups 2 and 4 that contain SpyCatcher-M2e alone or presented on noro-VLPs, respectively (**Figure 3A**). Without adjuvant, neither preparation was able to generate anti-M2e antibodies (Heinimäki et al., 2022). Vaccine candidates in groups 2 and 4 both seem promising as M2e-based influenza vaccines, as both generate equal anti-M2e antibody levels. Based on anti-SpyCatcher antibodies found in human serum samples (Rahikainen et al., 2021), we speculate that response against SpyCatcher could also produce some protection against the pathogenic bacterium *S. pyogenes*. Using a longer linker as described above could increase conjugation rates with SpyCatcher-M2e, thus increasing M2e/noro-VLP ratio and M2e density on particle surface in the vaccine candidate, which may be essential for more effective anti-M2e responses to occur. SpyCatcher-M2e would be simpler to manufacture, but on the other hand, vaccines containing noro-VLP generate substantial responses against norovirus, adding another clinical benefit.

Generating a strong response against the well-conserved M2e is great for an efficient influenza vaccine, but alone, it is not enough for protecting against influenza infections. The ectodomain of M2 protrudes only slightly out of the virion membrane and the concentration of M2 in an influenza virion is low (Zebedee and Lamb, 1988). Therefore, anti-M2e vaccines should be combined with a neutralizing influenza vaccine to prevent infection altogether. Anti-M2e response is most useful when cells have already been infected — e.g., because a virus strain has drifted too far from the one used in the neutralizing vaccine component to be completely neutralized. Cells infected with influenza virus express the M2 proton channel vigorously on their membrane to adjust their pH, which makes them easy targets for a broadly armed immune response against M2e (Hashemi et al., 2012). The M2e antibodies can aid in clearance of infected cells through antibody-enhanced natural killer cell or macrophage cytotoxicity, Fc opsonization or possibly prevent budding of new virions (Lee et al., 2019). In the case of influenza virus, the complement system is not only an antibody-dependent mediator of these protective mechanisms, but has been reported to be required for protection mediated by anti-M2e vaccines (Kim et al., 2018).

Prevention of serious influenza infection has been shown in challenge studies in the mouse and the ferret model (Neirynck et al., 1999; Fan et al., 2004; Tissot et al., 2010). A thorough, long-lasting protection against multiple strains of influenza virus and also against norovirus could be achieved by combining the most successful candidates in this study with an HA stem vaccine (Lampinen et al., 2021). Effective neutralizing effect against current strains can be added by mixing these with a commercial influenza vaccine, like Flublok (Sanofi Pasteur, France). Challenge studies should be conducted to test the *in vivo* protection capabilities of the influenza vaccine candidates reported here.

To our knowledge, immune responses against the SpyTag component have not been measured in earlier preclinical studies using the SpyCatcher/Tag conjugation system. Here, we measured a mild antibody response against SpyTag (the original version published in (Zakeri et al., 2012)) in vaccine group 4 containing SpyCatcher, noro-VLP and Al(OH)_3_. Surprisingly, SpyTag-noro-VLP alone was weakest in inducing anti-SpyTag antibodies, suggesting that incorporation into the SpyCatcher enhances the immunogenicity of SpyTag. As SpyTag is only 13 amino acid residues long, we bound biotinylated SpyTag on avidin for presentation to serum antibodies in ELISA. This approach proved essential for making the antigen available, as direct coating of the peptide on wells yielded no signal at all.

The clearance of influenza infection depends on the cooperation of CD4^+^ helper T lymphocytes, CD8^+^ cytotoxic T lymphocytes, and antibody-producing B lymphocytes (Heer et al., 2008). Therefore, vaccination should also induce cellular immune responses, instead of relying exclusively on antibody-mediated responses. In this study, we observed that even though the adjuvanted SpyCatcher-M2e (G2) and noro-VLP+SpyCatcher-M2e (G4) groups were effective in raising anti-M2e antibodies, these groups did not raise very strong cellular responses against M2e. In comparison, the vaccine group that received noro-VLP+SpyCatcher-M2e without Al(OH)_3_ elicited more robust cellular responses. This finding seems to demonstrate that Al(OH)_3_ can boost the antibody response with the expense of the cellular immune response. Future optimization of used adjuvant would be needed for a more balanced antibody and cellular mediated immune response. The commercial adjuvant system 04 (AS04) which consists of Al(OH)_3_ together with monophosphoryl lipid A (MPLA) could be considered in future experiments. MPLA has been shown to act as a TLR4 agonist and to promote IFN-γ production by antigen-specific CD4+ T cells, skewing the immune response toward a Th1 type direction (Casella and Mitchell, 2008; Didierlaurent et al., 2009).

In conclusion, our study demonstrates that display of influenza M2e on noro-VLP enhances its immunogenic capacity. Surprisingly, direct SpyCatcher-M2e fusion appears efficient in boosting the immunization against M2e. We, however, observed that conjugation of SpyCatcher-M2e on SpyTag-noro-VLP was beneficial for cellular immune responses in mice. Overall, the modular SpyTag-noro-VLP system appears a robust platform for optimization of experimental vaccine compositions.

## 4 Materials and methods

### 4.1 Preparation of vaccine antigens

Norovirus-like particle displaying C-terminal SpyTag (only original SpyTag version, published in Zakeri et al., 2012, was used in this study; SpyTag-noro-VLP: Addgene plasmid #165989) was produced as described previously (Lampinen et al., 2021), but for the removal of residual baculovirus, we used size-exclusion chromatography (SEC) instead of anion exchange chromatography. The norovirus VP1 gene was from norovirus strain Hu/GII.4/Sydney/NSW0514/2012/AU (GenBank accession no. AFV08795). Briefly, baculovirus (*Autographa californica* multiple nucleopolyhedrovirus, genome bMON14272 from DH10Bac (Thermo Fisher Scientific, USA, #10361012)) containing the target gene was amplified in Sf9 cells (Thermo Fisher Scientific, #11496015) and subsequently used to infect High Five insect cells (Thermo Fisher Scientific, #B85502). 4–6 days after infecting insect cells with baculovirus, the SpyTag-noro-VLP was concentrated from the supernatant by ultracentrifugation (175 000 g, 6–16 h) through a sucrose cushion. After ultracentrifugation, residual baculovirus was removed by SEC using the ÄKTA Purifier instrument and HiPrep 16/60 Sephacryl S-500 HR column (Cytiva, USA, #28935606). SpyCatcher-M2e (Only original SpyCatcher version, published in Zakeri et al., 2012, was used in this study; M2e sequence according to consensus of human influenza sequences, MSLLTEVETPIRNEWGCRCNDSSD; Addgene plasmid #165990) was expressed in *E. coli* as explained in (Lampinen et al., 2021) and conjugated on SpyTag-noro-VLP by adding a 2-fold molar excess of SpyCatcher-M2e and incubating overnight. Excess SpyCatcher-M2e was removed by another round of SEC, using the setting described above, instead of dialysis. The same M2e sequence was used in all preparations in this study.

For production of direct fusion between norovirus VP1 and M2e, SpyTag in the 3’ end of SpyTag-noro-VLP gene was replaced by influenza M2e (Addgene plasmid #201192) and the expression cassette was subcloned into pOET5.1 vector (Oxford Expression Technologies, UK, #200106), under the polyhedrin late promoter by GenScript (USA). In the expression construct, M2e peptide is separated from the norovirus VP1 C-terminus by a 4-amino-acid linker (TSGG). M2e-noro-VLP was produced and purified as described above for SpyTag-noro-VLP, except that we used FlashBAC ULTRA baculovirus genome (Oxford Expression Technologies, UK, #100150) to produce the baculovirus. The M2e (Proteogenix, France) and SpyTag (AHIVMVDAYKPTK, original version published in Zakeri et al., 2012) peptides used for ELISA coating and splenocyte stimulation were chemically synthesized and purified to >85% purity by GenScript. The SpyTag peptide used for splenocyte stimulation was cleared of endotoxins by GenScript.

### 4.2 Characterization of recombinant protein and nanoparticle antigens

Protein purity and conjugation efficiency were estimated by densitometric analysis of Any kD Stain-free SDS-PAGE gels (#4568126 and #5678125) with the Image Lab software (Bio-Rad, USA). Conjugation efficiency was defined as the densitometric weight of noro-VP1/SpyCatcher conjugate divided by the weight of all noro-VP1 bands. We used the PageRuler Unstained Broad Range Protein Ladder (Thermo Fisher, #26630) as molecular weight marker. For Western blotting, we transferred the gel-separated proteins onto nitrocellulose membrane using Trans-blot Turbo (Bio-Rad). The presence of M2e was confirmed with an anti-influenza-M2 antibody (1:3000, Thermo Fisher, #ma1-082). A mouse monoclonal anti-gp64 antibody (1:2000, Santa Cruz Biotechnology, USA, #sc-65499) was used to verify the absence of residual baculovirus in the purified noro-VLPs. The bound primary antibodies were visualized by IRDye 800CW goat anti-mouse IgG secondary antibody (1:20 000, LI-COR Biosciences, USA, #926–32210) and the Odyssey CLx instrument (LI-COR Biosciences, USA). We measured protein concentrations using the Pierce BCA protein assay (Thermo Fisher Scientific, #23252). Endotoxin concentrations were determined with ToxinSensor Chromogenic LAL Endotoxin Assay Kit (GenScript, #L00350) and the amount of residual DNA was measured with the Quant-iT dsDNA high sensitivity kit (Thermo Fisher Scientific, #Q33120).

We used dynamic light scattering (DLS) analysis with the Zetasizer Nano ZS (Malvern Instruments, UK) to measure the size and polydispersity of produced nanoparticles and proteins. F200 S/TEM (Jeol, Japan) transmission electron microscope was used to examine the morphology of noro-M2e after negative staining with 1% uranyl acetate. We compared the thermal stability of noro-M2e to that of SpyTag-noro-VLP with differential scanning fluorimetry (DSF), as described in detail elsewhere (Niesen et al., 2007). The DSF analyses were performed in phosphate buffer with different salt concentrations (50 mM Na_2_HPO_4_/NaH_2_PO_4_, 10/100/250 mM NaCl, pH 7.2), always using 4 µg VLP per reaction.

We used the VP-Capillary differential scanning calorimetry (DSC) instrument (GE Healthcare, USA) to measure the disassembly and unfolding of wild type noro-VLP at a concentration of 0.2 mg/mL in Tris-buffered saline (50 mM Tris-Cl, 150 mM NaCl, pH 7.4). We tried the same concentration for M2e-noro-VLP but were not able to get detectable unfolding signal before we increased concentration to 1.8 mg/mL, here dissolved in phosphate-buffered saline (PBS: 10 mM Na_2_HPO_4_, 1.8 mM KH_2_PO_4_, 137 mM NaCl, 2.7 mM KCl, pH 7.4). In all DSC measurements, we heated the samples from 20 to 110°C at a rate of 2°C/min. Feedback mode was set to “None”, and the filter period to 5 s. The Tm values were obtained from the midpoints of peak curves obtained by subtracting the buffer measurement baseline from measurement data and then fitting the curve with the Levenberg-Marquardt non-linear least-squares method. Data analysis was done using the MicroCal Origin 7.0 software (Malvern Instruments, UK). The results are averaged from two independent measurements.

### 4.3 Animal experiments

To evaluate the immunogenicity of the antigens, we randomly divided specific pathogen-free female BALB/cJRj mice (Janvier Labs, France) into groups of five animals (**Table 1**). Only female mice were used due to them being better adapted to group housing than male mice. In a previous study (Xia et al., 2011), the response had a standard deviation of 25 000 (unitless). If the true difference in the experimental and control antibody titer means is 50 000 in groups of 5 mice each, we would be able to reject the null hypothesis that the population means of the experimental and control groups are equal with a probability (power) of 0.85. The Type I error probability associated with this test is 0.05. The mice were acclimatized for a week before the first immunization (day 0), at which point they were 6 weeks old. The mice received a subcutaneous injection of 150 µL interscapularly at days 0 and 21. M2e doses were matched for each group according to **Table 1**, considering the size of M2e peptide compared to its carrier protein(s) and conjugation efficiency. An example of calculating M2e dose in a vaccine preparation for group 4 is provided below. M(SpyTag-noro-VLP)=61.01 kDa, M(SpyCatcher)=12.04 kDa, M(M2e)=2.76 kDa and conjugation efficiency was 24%.


$$24\%*31\text{μg}*\frac{2.76\text{ kDa}}{(61.01+12.04+2.76)\text{ kDa}}=0.27\text{μg}$$

We used “Alhydrogel adjuvant 2%” (Invivogen, USA, #vac-alu-250) Al(OH)_3_ as an adjuvant in the vaccination groups indicated in **Table 1**. In the adjuvanted groups, we added 100 µg of Al(OH)_3_ per dose.

We collected blood samples from tail veins at days 0, 21, 33 and 36 under inhalation anesthesia by isoflurane (Attane vet, Vet Medic Animal Health, Finland, #AP/DRUGS/220/96). At sacrifice on day 42, we collected whole blood by heart puncture and separated the serum with blood collection tubes (Thermo Fisher Scientific, #365968). We also collected spleens and extracted the splenocytes as described in (González-Rodríguez et al., 2022). The pre-clinical experiments were executed in accordance with the regulations and guidelines of the Finnish National Experiment Board (Permission number ESAVI/1408/2021). All efforts were made to minimize animal suffering and to reduce the number of animals used. The welfare of the animals was monitored throughout the experiment and Animal Research: Reporting of *In Vivo* Experiments (ARRIVE) guidelines were followed. Laboratory animal usage permission (Regional State Administrative Agency, Pirkanmaa, Finland; decision number ESAVI/1408/2021) covers all mouse experiments described here.

### 4.4 Serum analyses

The total IgG antibody levels against influenza M2e, noro-VLP and SpyTag were assessed with enzyme-linked immunosorbent assays (ELISA) from mouse serum samples, as described earlier (Blazevic et al., 2011). Briefly, Maxisorp 96-well-plates (Thermo Fisher Scientific, #439454) were coated with 50 ng of M2e peptide, SpyTag-noro-VLP or SpyCatcher protein fused with an unrelated peptide per well. For measuring anti-SpyTag antibodies, we coated the wells first with 250 ng of in-house wild type avidin (expressed recombinantly in *E. coli*) and then attached biotinylated SpyTag on the avidin for better availability of the peptide. After blocking the wells with bovine serum albumin and adding the serially diluted mouse sera from the immunization experiment, antigen-bound IgG antibodies in the sera were detected with horseradish peroxidase -conjugated horse anti-mouse monoclonal antibody (mAb) (1:4000, Vector, USA, #PI-2000) and OPD substrate (Merck, USA, #P8412). We used an in-house anti-noro-VLP mouse antiserum, a monoclonal mouse anti-His antibody (1:160 000, Thermo Fisher Scientific, #ma1-21315) and a monoclonal mouse anti-M2 antibody (1:600 000, Thermo Fisher, #ma1-082) as positive controls. Optical densities at 490 nm (OD490) were measured with a microplate reader (Victor Nivo, PerkinElmer, USA). Endpoint titers were defined as the reciprocal of the highest serum dilution with an OD490 above the positivity cut-off value. The positive cut-off value was defined as the (mean absorbance) +4.105*(standard deviation) of buffer+Al(OH)_3_ group sera at dilution 1:400. Multiplication with 4.105 gives a confidence level of 99% with 5 mice in the negative control group (Frey et al., 1998). All ELISA analyses were performed in duplicate and at least two independent experiments were performed, so all endpoint titers in this experiment are estimated from averages of at least four measurements.

### 4.5 Immune cell analyses

Cryopreserved splenocytes from vaccinated mice were thawed in splenocyte incubation medium consisting of RPMI 1640 Medium supplemented with GlutaMAX (Thermo Fisher Scientific, #61870-010), 500 U Penicillin-Streptomycin (Sigma-Aldrich, #P0781), 10% FBS (Sigma-Aldrich, #F9665), and 25 mM HEPES (Sigma-Aldrich, #H0887) and rested for 1 h at 37°C in a humified incubator with 5% CO_2_.

Simultaneous secretion of IFN-γ, IL-2, and TNF-α was analyzed with Mouse IFN-γ/IL-2/TNF-α FluoroSpot^PLUS^ kit (Mabtech, Sweden, #FSP-414245-10). We set up the assay in duplicate under sterile conditions and tested the splenocytes of individual mice separately according to the manufacturer’s instructions. Briefly, we washed plates pre-coated with mAbs AN18, 1A12, and MT1C8/23C9 from the kit with sterile PBS and blocked the plates with splenocyte incubation medium. After blocking, we added 1 µg of stimulant (antigen) diluted in splenocyte incubation medium together with 250 000 splenocytes to each well and incubated the plates at 37°C in a humified incubator with 5% CO_2_ overnight. Concanavalin A (Sigma-Aldrich, #C5275) (2 µg/well) and splenocyte incubation medium were used as positive and negative controls, respectively. Additionally, anti-CD28 mAb (1:1000) from the kit was used as a co-stimulator in each well to enhance antigen-specific responses, as recommended by the manufacturer. After the overnight incubation, we removed the cells by washing the plates with sterile PBS. We diluted the detection antibodies in PBS containing 0.1% bovine serum albumin (BSA) and added the BAM-tagged anti-IFN-γ mAb (1:200), biotinylated anti-IL-2 mAb (1:500), and WASP-tagged anti-TNF-α mAb (1:200) from the kit to the plates. The plates were then incubated for 2 hours at room temperature (RT) and washed with sterile PBS. Subsequently, anti-BAM-490, Streptavidin-550, and anti-WASP-640 fluorophore conjugates (diluted 1:200 in PBS-0.1% BSA) were added to the plates, which were incubated for 1 hour at RT. Then, we washed the plates with sterile PBS and added Fluorescence enhancer. After a 15-minute incubation at RT, we removed the Fluorescence enhancer and the bottom seals from the plates and dried the plates. Plates were then shipped to Mabtech for automated spot analysis with Mabtech IRIS FluoroSpot reader and the numbers of cytokine-secreting cells specifically activated by a stimulant were received as a readout. For each mouse and stimulant used, we calculated an average of the duplicate wells and subtracted the positivity cut-off value. We defined the positivity cut-off value for each mouse separately as the average number of spots in the negative control wells +3*(standard deviation). The final frequencies of responding cells were expressed as the number of spot-forming units/10^6^ splenocytes.

### 4.6 Statistical analyses

For statistical analyses, we used GraphPad Prism version 8.3.0 and defined that p<0.05 indicates statistically significant difference. To estimate differences in mean end-point titers and mean spot counts between vaccine groups and the negative control group, we used Kruskal-Wallis followed by Dunnett’s test. For the ELISA data, we used the corresponding mouse serum at day 0 as the control group, whereas the buffer immunized group served as a negative control for FluoroSpot data. We also compared the means of each group to all other groups with the Dunn’s test.

## Data availability statement

The raw data supporting the conclusions of this article will be made available by the authors, without undue reservation.

## Ethics statement

The animal study was reviewed and approved by the Finnish National Experiment Board. Laboratory animal usage permission (Regional State Administrative Agency, Pirkanmaa, Finland; decision number ESAVI/1408/2021) covers all mouse experiments described here.

## Author contributions

VL, MH, VH and VB contributed to conception and design of the study. VL produced the vaccines and performed most experiments in the supervision of MH and VH. SG and SS participated in the animal experiment, performed the cellular immunology assays and analyzed their results. VL drafted the first manuscript draft and figures, while SG wrote sections of the manuscript. All authors discussed the results and commented on the manuscript to help shape its final version. All authors read and approved the final manuscript.
