# Supplementary material for: SpyTag/SpyCatcher display of influenza M2e peptide on norovirus-like particle provides stronger immunization than direct genetic fusion
Source: Front Cell Infect Microbiol. 2023 Jun 22;13:1216364. doi: 10.3389/fcimb.2023.1216364 (PMC10323135; doi:10.3389/fcimb.2023.1216364)
Supplement: Supplementary file 1 [file DataSheet_1.docx]

Supplementary Material

SpyCatcher-mediated influenza M2e peptide display is more immunogenic than genetic fusion on the norovirus-like particle

Vili Lampinen, Stina Gröhn, Saana Soppela, Vesna Blazevic, Vesa Hytönen^†*^, Minna Hankaniemi^†*^

† These authors contributed equally to this work and share last authorship

*** Correspondence:**Minna Hankaniemi
[minna.hankaniemi@tuni.fi](mailto:minna.hankaniemi@tuni.fi)

Vesa Hytönen
[vesa.hytonen@tuni.fi](mailto:vesa.hytonen@tuni.fi)

# Supplementary Figures


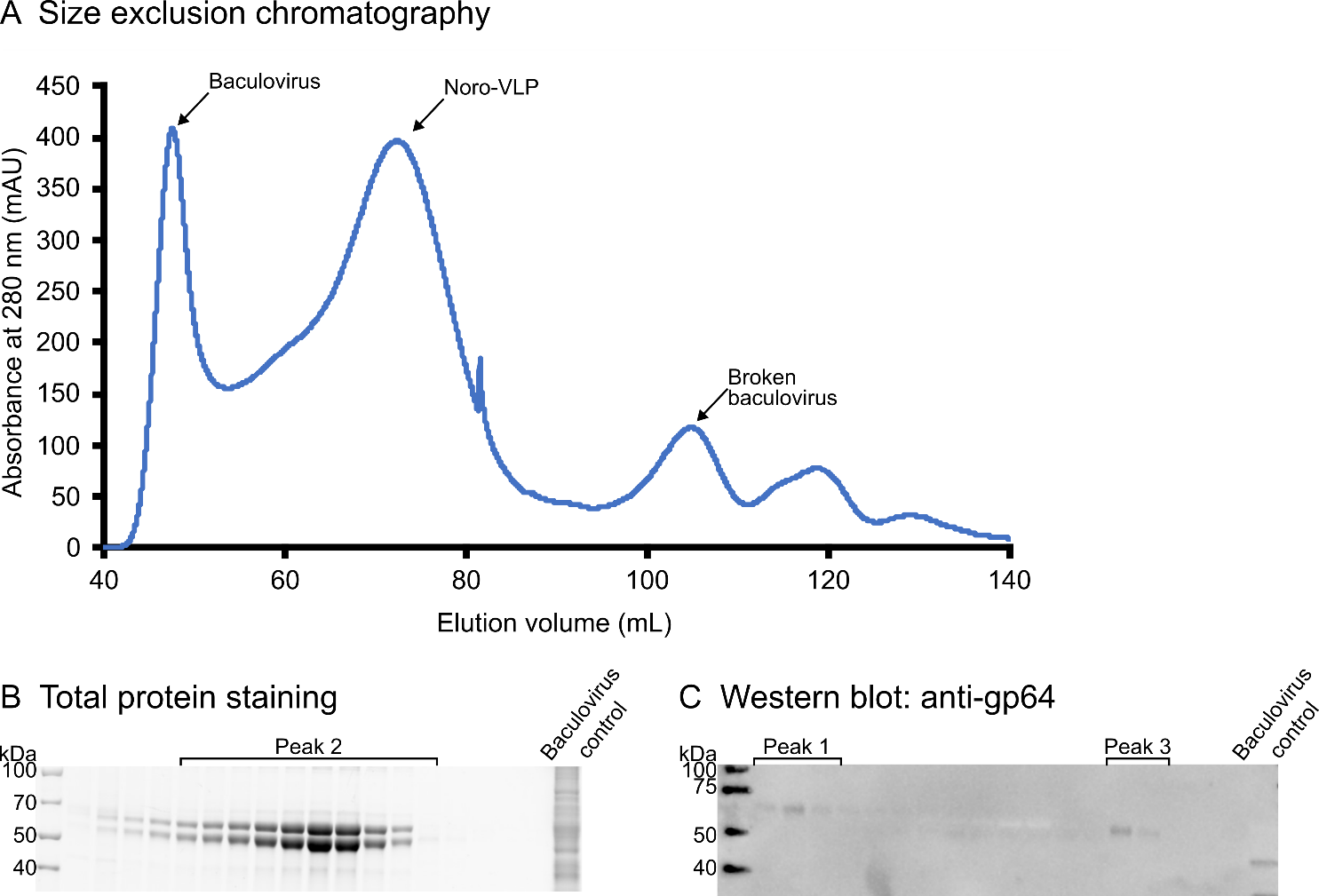


**Supplementary Figure 1.** **(A)** Chromatogram of a representative size exclusion chromatography run showing the peaks identified from fraction samples analyzed by **(B)** total protein staining and **(C)** anti-baculovirus gp64 protein Western blotting.


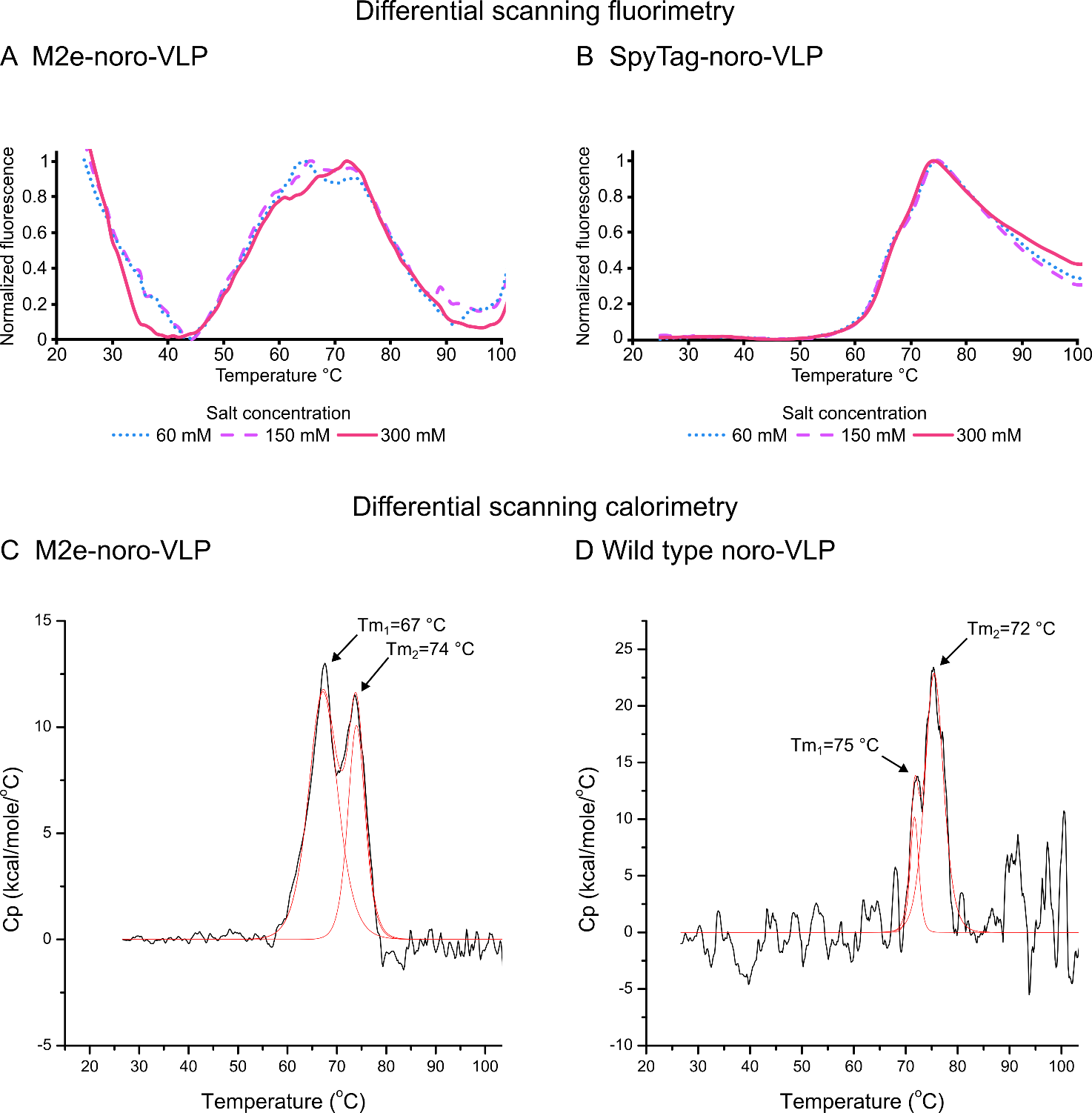


**Supplementary Figure 2.** Differential scanning fluorimetry analysis of M2e-noro-VLP **(A)** and SpyTag-noro-VLP **(B)** in 60, 150 or 300 mM total salt concentration. Each graph here is averaged from three independent measurements. Differential scanning calorimetry analysis of M2e-noro-VLP **(C)** and wild type noro-VLP **(D)**. The DSC curves were fitted with Levenberg-Marquardt non-linear least-squares method.

**
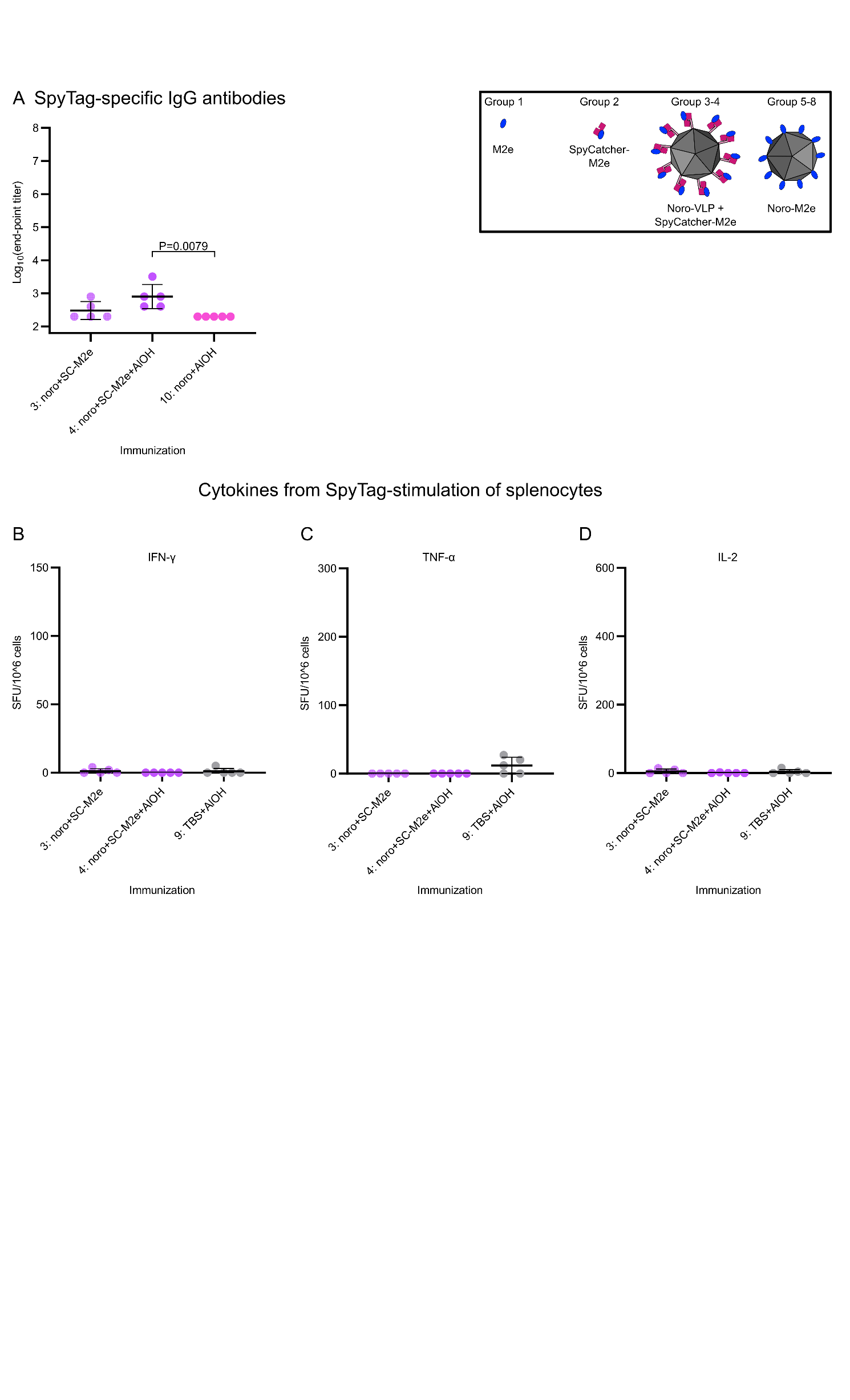
**

**Supplementary Figure 3.** **(A)** Log10 transformations of IgG antibody end-point titers against SpyTag, as measured in ELISA wells coated with the biotinylated peptide, presented on avidin. Undetectable antibody levels were denoted with the titer 200 (half of the lowest dilution measured). Interferon gamma **(B)**, tumor necrosis factor alpha **(C)** and interleukin-2 **(D)** levels obtained by stimulating splenocytes extracted from vaccinated mice with the SpyTag peptide. Mean titers and mean positive cell counts are presented as thick lines ± standard deviation. P values between groups are shown for pairs with a difference in means with P<0.05, determined by Dunn’s test. Each dot represents a single mouse.

**
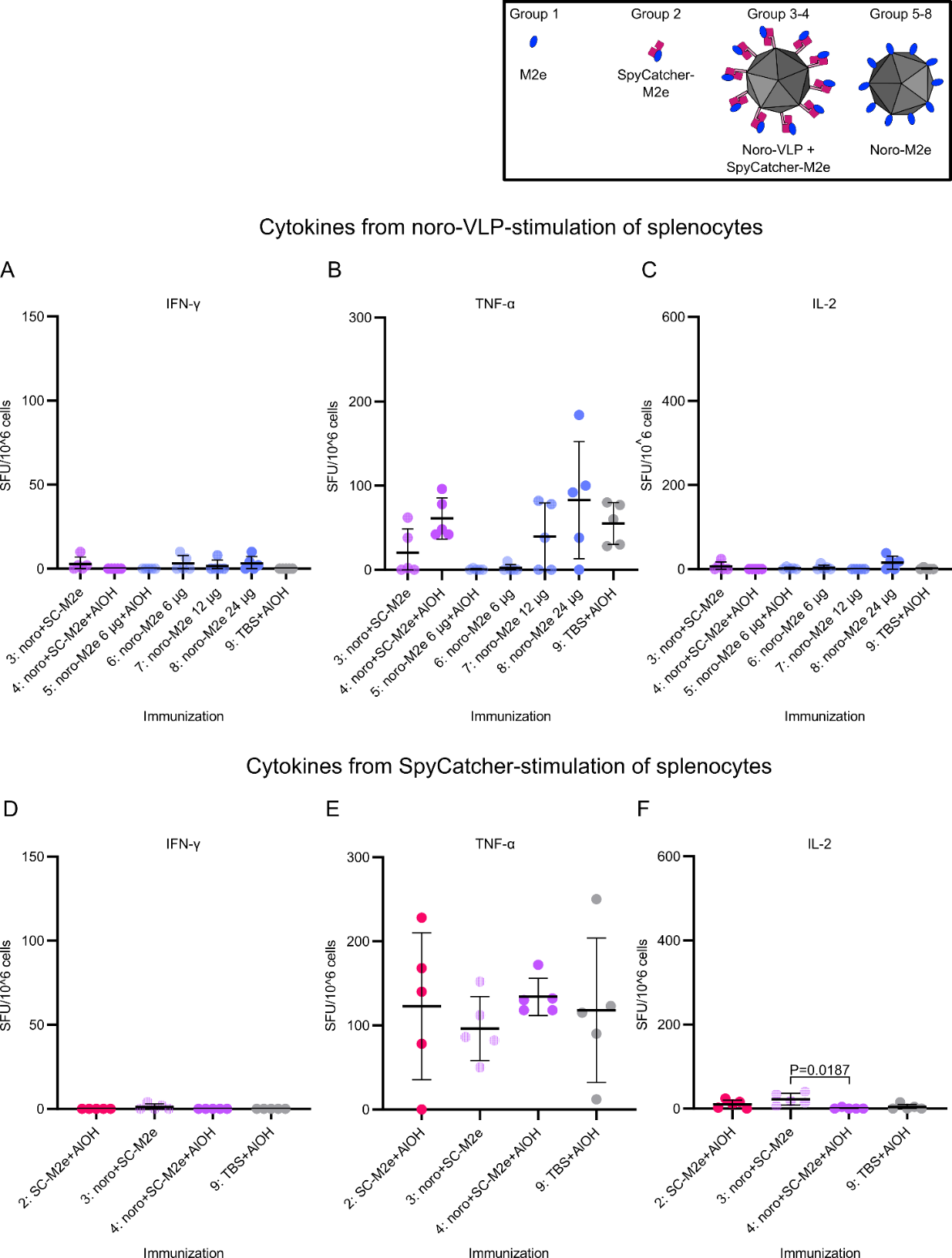
**

**Supplementary Figure 4.** Interferon gamma **(A, D)**, tumor necrosis factor alpha **(B, E)** and interleukin-2 **(C, F)** levels obtained by stimulating splenocytes extracted from vaccinated mice with noro-VLP **(A–C)** or SpyCatcher **(D–F)**. Mean positive cell counts are presented as thick lines ± standard deviation. P values between groups are shown for pairs with a difference in means with P<0.05, determined by Dunn’s test. Each dot represents a single mouse.
